# Factors associated with chronic venous disease: study in 1,136 patients treated for varicose veins of the lower limbs in a specialized clinic

**DOI:** 10.1590/1677-5449.202200513

**Published:** 2022-10-10

**Authors:** Martha Ofelia Correa Posada, Laura Maria Contreras Correa, John Fernando García Vélez

**Affiliations:** 1 Universidad de Antioquia (U de A), Medellín, Antioquia, Colombia.; 2 Universidad CES (U CES), Medellín, Antioquia, Colombia.; 3 General Practitioner in Vía Vascular Medical, Medellín, Antioquia, Colombia.

**Keywords:** varicose veins, epidemiology, risk factors

## Abstract

**Background:**

Varicose veins are a highly prevalent condition in the general population, generating variable reasons for consultation that can alter the patient’s quality of life, with prevalence and associated factors that vary in different series.

**Objectives:**

To describe the epidemiological profile of patients who consulted for varicose veins by evaluating main symptoms and associated variables.

**Methods:**

Between 2019 and 2020, 1,136 patients attending vascular surgery consultation in a specialized outpatient center were evaluated. Demographic variables, presented symptoms, complications, and associated factors, such as body mass index, parity and family history, were recorded.

**Results:**

A total of 1136 patients were evaluated (79.8% women and 20.2% men), with a mean age of 53.51 years. The presence of symptoms was similar in men and women; the most frequent complications were ulcer, varicorrhage, and superficial venous thrombosis. Most patients showed CEAP 1, 2 and 3 (n = 909) and more than half were overweight or obese (n = 679) with a predominance of those classified as C4. Sixty-nine per cent had a positive family history of varicose veins. There was no difference between severity of varicose veins and time working in the standing or sitting position, but there was a greater presence of C5 or C6 ulcer among patients standing for more than 4 hours.

**Conclusions:**

Describing the characteristics of patients with varicose veins helps to understand the disease and to focus efforts towards those who are more susceptible. The results of this research are similar to those found in other populations.

## Resumo

**Contexto:**

As varizes são uma condição altamente prevalente na população em geral, gerando motivos variáveis de consulta que podem alterar a qualidade de vida do paciente, com prevalência e fatores associados variando em diferentes séries.

**Objetivos:**

Descrever o perfil epidemiológico dos pacientes que consultaram por varizes, avaliando os principais sintomas e as variáveis associadas.

**Métodos:**

Entre 2019 e 2020, foram avaliados 1.136 pacientes que compareceram a uma consulta de cirurgia vascular em um centro ambulatorial especializado. Foram registradas variáveis demográficas, apresentando sintomas, complicações e fatores associados tais como índice de massa corporal, paridade e histórico familiar.

**Resultados:**

Foram avaliados 1.136 pacientes (79,8% mulheres e 20,2% homens), com idade média de 53,51 anos. A presença de sintomas foi similar em homens e mulheres; as complicações mais freqüentes foram úlcera, varicorragia e trombose venosa superficial. A maioria dos pacientes apresentava CEAP 1, 2 ou 3 (n = 909), e mais da metade tinha excesso de peso ou obesidade (n = 679), com predominância dos classificados como C4. Sessenta e nove por cento tinham histórico familiar positivo de veias varicosas. Não houve diferença entre a gravidade das varizes e o tempo gasto em pé ou sentado, mas havia uma presença maior de úlcera C5 ou C6 em pacientes que permaneciam em pé por mais de 4 horas.

**Conclusões:**

A descrição das características da população com varizes ajuda a entender a doença e a canalizar os esforços para aqueles mais suscetíveis. Os resultados desta investigação são semelhantes aos encontrados em outras populações.

## INTRODUCTION

Varicose veins are a frequent condition in primary healthcare visits and one of the most treated by vascular surgeons worldwide, producing an array of important symptoms and long-term complications that have an impact on health and quality of life, which also increase health expenses.^1^ Knowledge on the profile of the population with varicose veins improves disease understanding and patient's education, thus helping to focus efforts on early care and having an influence on their outcome.^2^ The aim of this study was to present an epidemiological profile of patients who consulted for varicose veins, assessing their main symptoms and associated variables.

## METHODS

This is a prospective, descriptive study that collected data from patients who attended a vascular surgery consultation at a vascular disease center in the city of Medellin, Colombia, from June 2019 to December 2020. Patients who consulted for varicose veins were asked about variables related to venous disease, and their responses were inserted into a database previously developed on Excel during the consultation, including demographic variables, related medical history, symptoms, previous treatments, complication, family history, habits, and clinical variables such as severity according to the clinical-etiological-anatomical-pathophysiological (CEAP) classification and body mass index (BMI). Of note, confinement due to the COVID-19 pandemic was implemented in 2020, which slowed data collection down, and only face-to-face consultations that could be performed in this period were considered.

This study complied with guidelines on personal data management and with institutional ethical policies, being categorized as a non-hazardous investigation. Data were processed on SPSS software, version 22, using frequencies and percentages for categorical variables and mean and standard deviation (SD) for quantitative variables. The chi-squared was used to investigate the relationship between categorical variables, considering a statistical significance level of p < 0.05.

This study was conducted in compliance with the Declaration of Helsinki and the Colombian law on ethics and investigation: Resolution 8430 of 1993, issued by the Ministry of Health and Social Protection of Colombia. The protocol was approved by the ethics committee of the research institution (record number 002 of January 21st, 2019).

## RESULTS

Overall, 1,136 patients were evaluated, consisting of 911 women (80%) and 225 men (20%). Mean age was 53.51 years (SD±12.53; range 15-98 years) for the general population, 54 (SD±12.25) years for women, and 51.32 (SD±13.39) for men, with no age difference between the two groups (p = 0.127). The age distribution between genders and other characteristics are presented in Table 1.

**Table 1:** Variables associated with varicose veins according to gender.

**Variable**	**Women**		**Men**		**Total**	
	**n (911)**	**%**	**n (225)**	**%**	**n = 1,136 (%)**	**p-value**
**Age (years)**						0.045
15-35	76	8.3	31	13.8	107 (9.4)	
36-50	275	30.2	71	31.6	346 (30.5)	
51-60	282	31.0	68	30.2	350 (30.8)	
> 60	278	30.5	55	24.4	333 (29.3)	
**CEAP clinical classification**						0.000
0						
1	7	0.8	6	2.7	13 (1.1)	
2	217	23.8	15	6.7	232 (20.4)	
3	429	47.1	108	48.0	537 (47.3)	
4	161	17.6	38	16.9	199 (17.5)	
5	38	4.2	26	11.6	64 (5.6)	
6	39	4.3	22	9.8	61 (5.5)	
	20	2.2	10	4.4	30 (2.6)	
**Symptoms**						
Edema	433	47.5	94	41.8	527 (46.4)	0.121
Heaviness	419	46.0	86	38.2	505 (44.5)	0.36
Tiredness	563	61.8	125	55.6	688 (60.6)	0.086
Burning sensation	514	56.4	83	36.9	597 (52.6)	0.000
Itching	367	40.3	80	35.6	447 (39.3)	0.193
Pain	598	65.6	123	54.7	721 (63.5)	0.002
Cramps	402	44.1	86	38.2	488 (43.0)	0.196
**BMI ((kg/m^2^)**						0.103
Underweight (< 18.5)	6	0.7	4	1.8	10 (0.9)	
Normal weight (18.5–24.99)	322	35.3	77	34.2	399 (35.1)	
Overweight (25–29.99)	347	38.1	98	43.6	445 (39.2)	
Obesity (>30)	236	26.0	46	20.4	282 (24.8)	
**Associated factor**						
Diabetes	56	6.1	10	4.4	66 (5.8)	0.328
Hypothyroidism	166	18.2	8	3.6	174 (15.3)	0.000
Smoking	72	8.0	30	13.3	102 (9.0)	0.011
Constipation	134	14.7	10	4.4	144 (12.7)	0.000

CEAP = clinical-etiological-anatomical-pathophysiological; BMI = body mass index.

Furthermore, 891 (78.33%) patients were classified into grades from 2 to 6 of the CEAP clinical classification. C4 grade or higher was more frequent in men than in women, whereas C2 and C3 had a similar frequency in both sexes. The distribution of symptoms was similar between men and women, with difference only for the presence of ardor and pain, which predominated in women. Pain was the most reported symptom in both genders, followed by tiredness and ardor. Of the symptoms evaluated, there were gender differences only for burning sensation and pain, which predominated in women (p < 0.05).

The population studies had a low prevalence of diabetes, hypothyroidism, constipation, and smoking, but there was a remarkably high prevalence of overweight and obesity (64%) in this population. However, no differences in nutritional status were found between men and women; conversely, a higher presence of C4 grade or greater was observed in patients with BMI above 25.

Moreover, 69% (782 patients) had a family history of at least one parent with varicose veins, and 13.11% (149 patients) had both parents with varicose veins. Furthermore, 212 patients (19%) had developed one or more complications, the most common of which was ulcer, which was present in 91 patients (8%), followed by varicorrhage, present in 22 (2.1%). Also, 83 (7.9%) patients had a history of superficial venous thrombosis, 19 (1.08%) had a history of deep venous thrombosis, and 11 (1.04%) presented associated lymphedema. Only 2.6% had an active ulcer at the time of assessment.

No differences were found in symptom distribution between genders and among age groups, except for tiredness, which predominate in patients older than 35 years in both groups (women: p = 0.003; men: p = 0,011). In women, there were differences in weight distribution by age groups, with overweight and obesity predominating among those older than 35 years, whereas men exhibited a similar distribution for all age groups (Table 2).

**Table 2:** Distribution of symptoms and nutritional status according to age group.

**Symptom**	**Age group**				
	**15-35 years**	**36-50 years**	**51-60 years**	**>60 years**	**p-value**
**Women**					
Edema	39 (4.3%)	131 (14.4%)	136 (14.6%)	127 (13.9%)	0.832
Heaviness	43 (4.7%)	137 (15.0%)	118 (13.0%)	121 (13.3%)	0.053
Tiredness	61 (6.7%)	174 (19.1%)	166 (18.2%)	162 (17.8%)	0.003
Burning sensation	44 (4.8%)	156 (17.1%)	162 (17.8%)	152 (16.7%)	0.909
Itching	36 (4.0%)	116 (12.7%)	110 (12.2%)	105 (11.5%)	0.404
Pain	53 (5.8%)	176 (19.3%)	188 (20.6%)	181(19.9%)	0.787
Cramps	38 (4.2%)	124 (13.6%)	120 (13.2%)	120 (13.2%)	0.67
**Men**					
Edema	14 (6.2%)	32 (14.2)	26 (11.6%)	22 (9.8%)	0.829
Heaviness	15 (6.7%)	33 (14.7%)	22 (9.8%)	16 (87.1%)	0.096
Tiredness	23 (10.2%)	45 (20.0%)	34 (15.1%)	23 (10.2%)	0.011
Burning sensation	15 (6.7%)	26 (11.6%)	24 (10.7%)	18 (8.0%)	0.523
Itching	16 (7.1%)	26 (11.6%)	21 (9.3%)	17 (7.6%)	0.196
Pain	17 (7.6%)	41 (18.2%)	33 (14.7%)	32 (14.2%)	0.664
Cramps	12 (5.3%)	28 (12.4%)	25 (11.1%)	21 (9.3%)	0.991
**Women**					0.001
Underweight (< 18.5 kg/m^2^)	0 (0.0%)	0 (0.0%)	2 (0.2%)	4 (0.4%)	
Normal weight (18.5-24,9 kg/m^2^)	44 (4.8%)	106 (11.6%)	86 (9.4%)	86 (9.4%)	
Overweight (25-29,9 kg/m^2^)	18 (2.0%)	108 (11.9%)	113 (12.4%)	108 (11.9%)	
Obesity (> 30 kg/m^2^)	14 (1.5%)	61 (6.7%)	81 (8.9%)	80 (8.8%)	
**Men**					0.072
Underweight (< 18.5 kg/m^2^)	3 (1.3%)	0 (0.0%)	1 (0.4%)	0 (0.0%)	
Normal weight (18.5-24,9 kg/m^2^)	11 (4.9%)	24 (10.7%)	21 (9.3%)	21 (9.3%)	
Overweight (25-29,9 kg/m^2^)	12 (5.3%)	29 (12.9%)	32 (14.2%)	25 (11.1%)	
Obesity (> 30 kg/m^2^)	5 (2.2%)	18 (8.0%)	14 (6.2%)	9 (4.0%)	

With regard to distribution according to clinical severity, most patients with advanced disease were older than 50 years of age, and most patients attended consultation with C2, followed by C1, in all age groups (Table 3).

**Table 3:** Distribution of age and symptoms according to CEAP clinical classification.

**Variable**	**CEAP clinical classification**							
	**C0**	**C1**	**C2**	**C3**	**C4**	**C5**	**C6**	**P-value**
**Age (years)**								0.000
15-35	2 (1.9%)	14(13.1%)	65 (60.7%)	15 (14.0%)	5 (4.7%)	2 (1.9%)	4 (3.7%)	
36-50	4 (1.2%)	76 (22.0%)	164(47.4%)	72 (20.8%)	12 (3.5%)	11 (3.2%)	7 (2.0%)	
51-60	6 (1.7%)	75(21.4%)	163(43.9%)	64 (18.3%)	17 (4.9%)	16 (4.6%)	9 (2.6%)	
> 60	1 (0.3%)	67(20.1%)	145 (43.5%)	48 (14.4%)	30 (9.0%)	32 (9.6%)	10 (3.0%)	
**Symptoms**								
Edema	4 (0.8%)	82(15.6%)	213 (40.4%)	140 (26.6%)	35 (6.5%)	19 (3.6%)	1 (3.6%)	0.000
Heaviness	7 (1.4%)	101 (20.0%)	231 (45.7%)	99 (19.6%)	24 (4.8%)	26 (5.1%)	17 (3.4%)	0.380
Tiredness	8 (1.2%)	131 (19.0%)	339 (49.3%)	126 (18.3%)	32 (4.7%)	33 (4.8%)	19 (2.8%)	0.245
Burning sensation	7 (1.2%)	121(20.3%)	289 (48.4%)	(17.9.6%)	28 (4.7%)	21 (3.5%)	24 (4.0%)	0.003
Itching	3 (3.7%)	81 (18.1%)	206 (46.1%)	84 (18.8%)	32 (7.2%)	29 (6.5%)	12 (2.7%)	0.172
Pain	8 (1.1%)	137 (19.0%)	341(47.3%)	135 (18.7%)	42 (5.8%)	31 (4.3%)	27 (3.7%)	0.009
Cramps	0 (0.0)	102(20.9%)	237(48.6%)	92 (18.9%)	21 (4.3%)	20 (4.1%)	16 (3.3%)	0.007

CEAP = clinical-etiological-anatomical-pathophysiological.

Most symptomatic patients were classified into C2, with a predominance of pain, tiredness, and cramps in this group of patients. In patients with C0 and C6, the most prevalent symptoms were tiredness, heaviness, and burning sensation. Only burning sensation and pain presented statistically significant differences between the genders. Table 3 shows the relationships between CEAP clinical scores and factors associated with varicose veins.

Mean number of pregnancies was 2.72 (SD± 2.1). Ninety-one women (8%) had never been pregnant at the time of the study, 408 (36%) had from one to two pregnancies, and 412 women (36.3%) had more than three pregnancies. When comparing number of pregnancies with severity according to CEAP clinical classification, no statistically significant difference was found (p = 0.196). However, the number of women with advanced disease (C4 or greater) was higher in those who had three or more pregnancies (58 vs. 34) (Table 3).

To conclude, 575 (50.6%) patients had some paid work. The mean number of hours working in the sitting and standing positions was 4.92 (SD±2.49) and 6.85 (SD±1.88) hours, respectively, based on an 8-hour work day. When grouping work hours, no differences in severity were found between time working in the sitting and standing positions; although the presence of C5 or C6 ulcer was greater in those who remained in the standing position for more than 4 hours.

## DISCUSSION

This article is based on the results from the general consultation to vascular surgeons, which may be a limitation; however, it focused the results on factors associated with venous disease, an aspect useful in daily practice.

Although differences between men and women with varicose veins may vary depending on inclusion criteria and methods used in the different studies, most reports show a higher frequency in men than in women, as observed in this series (80% vs. 20%), which may consist of a selection bias, possibly reflecting the greater number of women consulting for varicose veins.^3^^-^5 Also, there was a greater proportion of women in the C1 stage compared with men (217 vs. 15), maybe due to the esthetic impact in women, which would increase the search for consultation in early stages. However, there was also a greater proportion of symptoms in women with C1 compared with men in the same category. Consistent with this series, other studies have shown a higher proportion of men with visible trophic changes and advanced disease.^6^^,^^7^

Several authors have shown disease progression over time,^8^^-^10 with a low prevalence in those younger than 30 years, consistent with data from this study, in which most patients were aged above 35 years in both genders. Furthermore, the number of patients classified as stages C4-C6 increases with age, which is in line with the fact that venous disease requires time to cause more severe damage and that early intervention may be necessary to reduce vascular health impairment.^11^

A wide range of symptoms are present in venous disease but are not specific of this entity, which sometimes makes differential diagnosis complex. An analysis of CEAP clinical classification showed that only 1.1% of patients were classified into C0, a stage in which symptoms may be present, but there are no objective signs of varicose veins, a very low percentage when compared with population-based studies such as the *Vein Consult*, in which the prevalence of C0 was 20%.^12^ This may be due to the fact that many patients who attend vascular consultations have already been previously assessed by primary healthcare physicians, who, in the absence of external signs of varicose veins, may not refer the patient to specialized care.

The Edinburgh study showed differences in symptoms such as edema, tiredness. and itching, which were more frequent in young women than in men.^13^ However, the present study found a very similar gender distribution between the different age groups, possibly due to the greater number of patients classified as C2 or greater, which may be associated with a greater presence of symptoms, and to delayed access to vascular surgery as a sub-specialty in the health system, which makes patients be more symptomatic when attending consultation.

Pregnancy was found to be a risk factor for the development of varicose veins, maybe due to hemodynamic changes secondary to increased intra-abdominal pressure or to the presence of high circulating levels.^14^ A meta-analysis aiming to determine whether a history of pregnancies is associated with the development of varicose veins found that the odds of developing varicose veins increases by 82% (odds ratio [OR] = 1.82; 95% confidence interval [95%CI] = 1.43-2.33) for women with a history of pregnancy compared with no history of pregnancy.^15^ Lee et al.^10^ evaluated factors associated with progression of venous disease in the population in the Edinburgh study did not found an association between disease severity and number of pregnancies. A prevalence study in the Colombian population from an indigenous reservation showed that age, female sex, parity (more than 4 pregnancies), fat in the thigh, and large size were associated with telangiectasias, but there was no association with more advanced stages of varicose veins.^16^ In the present series, no relationship was observed between number of pregnancies and disease severity.

Several reports have shown a family history of varicose veins among people affected, with percentages ranging from 42% to 85%, which is consistent with data from the present study, in which 69% of the patients had at least one of padres con afectación.^17^^-^19 Some studies were able to identify genes related to venous disease that can explain the influence of the presence of first-degree relatives with venous disorders.^20^^-^22

There is no consistency in the literature whether smoking plays an important role in the development of varicose veins. In line with the present study, some authors did not observed such relationship;^23^^,^^24^ conversely, Yuan et al. recently reported that smoking is an independent risk factor (OR = 2.53; 95%CI = 1.15–5.58),^25^ whereas other authors found that there may be a positive association between genetic predisposition to smoking initiation and risk for varicose veins (OR = 1.12; 95%CI = 1.04–1.22; p = 0.004).^26^ Based on Burkit's hypothesis on the association between intestinal transit time and different diseases, constipation emerges as a factor that some authors relate to the development of varicose veins, due to increased intra-abdominal pressure;^27^ however, the association with this factor could not be confirmed, since prevalence in this population was low.

Obesity has also shown to be related to a positive correlation between BMI and development and severity of varicose vein, with obese patients presenting greater severity, due to increased intra-abdominal pressure resulting from the mass effect of the fat contained within the abdomen compressing the veins of the pelvis and possibly impairing venous return, changes that may be applied to increased intra-abdominal pressure caused by uterine enlargement during pregnancy.^28^^-^30 This study found differences with regard to the presence of varicose veins with different C grades in terms of nutritional status, showing a higher proportion of active ulcer or history of venous ulcer in overweight or obese patients compared with non-obese ones (54% vs. 2.28%), findings similar to those from other series in which higher BMI was associated with the development of ulcers.^31^^,^^32^

The prevalence of active ulcer in the study population was 2.6%, which is consistent with most series.^4^^,^^33^ Varicorrhage is a complication that, if not properly managed, may even be fatal, especially when the patient did not receive proper first aid, being the cause of death in 0.01% of autopsies.^34^^-^36 The incidence of this complication is difficult to establish in the literature, possibly due to its low prevalence, as shown by the fact that, in this study, only 22 patients had experienced an episode of varicorrhage.

No relationship was found between time working sitting or standing and severity of varicose veins, although the present study did not consider the time of exposure to this factor or whether the position was static or dynamic. Lack of uniformity in these criteria among the different studies made it difficult to find a relationship between occupation and development of varicose veins, in addition to the presence of multiple factors that may have an impact on the development of this condition. However, some reports suggest that remaining in one position for a prolonged time may be positively associated with the development of varicose veins and that positive changes in lifestyle may help reduce the severity of symptoms and improve work-related quality of life.^37^^-^39

In conclusion, the results of this study that the distribution of venous disease in the assessed population is very similar to that found in other series, support the progression of clinical severity with age and BMI, and indicate a higher frequency of family history of varicose veins in the evaluated patients. Knowledge of population's characteristics favors guidance on the natural disease history to patients and also improves resource optimization by establishing priorities based on the existence of risk factors or derived complications, which may lead to improved quality of life and reduced health expenditure.

## References

[1] Lumley E, Phillips P, Aber A, Buckley-Woods H, Jones GL, Michaels JA (2019). Experiences of living with varicose veins: a systematic review of qualitative research. J Clin Nurs.

[2] Youn YJ, Lee J (2019). Chronic venous insufficiency and varicose veins of the lower extremities. Korean J Intern Med (Korean Assoc Intern Med).

[3] Beebe-Dimmer JL, Pfeifer JR, Engle JS, Schottenfeld D (2005). The epidemiology of chronic venous insufficiency and varicose veins. Ann Epidemiol.

[4] Nicolaides A, Kakkos S, Baekgaard N (2018). Management of chronic venous disorders of the lower limbs. Guidelines According to Scientific Evidence. Part I. Int Angiol.

[5] Lins EM, Barros JW, Appolônio F, Lima EC, Barbosa M, Anacleto E (2012). Perfil epidemiológico de pacientes submetidos a tratamento cirúrgico de varizes de membros inferiores. J Vasc Bras.

[6] Criqui MH, Jamosmos M, Fronek A (2003). Chronic venous disease in an ethnically diverse population: the San Diego Population Study. Am J Epidemiol.

[7] Chiesa R, Marone EM, Limoni C, Volontè M, Petrini O (2007). Chronic venous disorders: Correlation between visible signs, symptoms, and presence of functional disease. J Vasc Surg.

[8] Brand FN, Dannenberg AL, Abbott RD, Kannel WB (1988). The epidemiology of varicose veins: the Framingham Study. Am J Prev Med.

[9] Brand FN, Dannenberg AL, Abbott RD, Kannel WB (1988). The epidemiology of varicose veins: the Framingham Study. Am J Prev Med.

[10] Lee AJ, Robertson LA, Boghossian SM (2015). Progression of varicose veins and chronic venous insufficiency in the general population in the Edinburgh Vein Study. J Vasc Surg Venous Lymphat Disord.

[11] Labropoulos N (2019). How does chronic venous disease progress from the first symptoms to the advanced stages? A review. Adv Ther.

[12] Rabe E, Guex JJ, Puskas A, Scuderi A, Fernandez Quesada F, VCP Coordinators (2012). Epidemiology of chronic venous disorders in geographically diverse populations: results from the Vein Consult Program. Int Angiol..

[13] Bradbury A, Evans C, Allan P, Lee A, Ruckley CV, Fowkes FGR (1999). What are the symptoms of varicose veins? Edinburgh vein study cross sectional population survey. BMJ.

[14] Barros N, Janeiro Perez MC, de Armori JE, Miranda F (2010). Pregnancy and lower limb varicose veins: prevalence and risk factors. J Vasc Bras..

[15] Ismail L, Normahani P, Standfield NJ, Jaffer U (2016). A systematic review and meta-analysis of the risk for development of varicose veins in women with a history of pregnancy. J Vasc Surg Venous Lymphat Disord.

[16] García-Pineda AF, Duque-Botero J, Cardona-Arias JA (2019). Epidemiología de los desórdenes venosos crónicos y factores asociados en amerindios nativos embera-chamí, Antioquia. Rev Fac Nac Salud Pública.

[17] Anwar MA, Georgiadis KA, Shalhoub J, Lim CS, Gohel MS, Davies AH (2012). A review of familial, genetic, and congenital aspects of primary varicose vein disease. Circ Cardiovasc Genet.

[18] Hirai M, Naiki K, Nakayama R (1990). Prevalence and risk factors of varicose veins in Japanese Women. Angiology.

[19] Scott TE, LaMorte WW, Gorin DR, Menzoian JO (1995). Risk factors for chronic venous insufficiency: a dual case-control study. J Vasc Surg.

[20] Fukaya E, Flores AM, Lindholm D (2018). Clinical and Genetic Determinants of Varicose Veins. Circulation.

[21] Krysa J, Jones GT, Van Rij AM (2012). Evidence for a genetic role in varicose veins and chronic venous insufficiency. Phlebology.

[22] Shadrina AS, Sharapov SZ, Shashkova TI, Tsepilov YA (2019). Varicose veins of lower extremities: insights from the first large-scale genetic study. PLoS Genet.

[23] Selçuk Kapısız N, Uzun Kulaoğlu T, Fen T, Kapısız HF (2014). Potential risk factors for varicose veins with superficial venous reflux. Int J Vasc Med.

[24] Beebe-Dimmer JL, Pfeifer JR, Engle JS, Schottenfeld D (2005). The epidemiology of chronic venous insufficiency and varicose veins. Ann Epidemiol.

[25] Elamrawy S, Darwish I, Moustafa S, Elshaer N, Ahmed N (2021). Epidemiological, life style, and occupational factors associated with lower limb varicose veins: a case control study. J Egypt Public Health Assoc.

[26] Yuan S, Bruzelius M, Damrauer SM, Larsson SC (2021). Cardiometabolic, Lifestyle, and Nutritional Factors in Relation to Varicose Veins: A Mendelian Randomization Study. J Am Heart Assoc.

[27] O’Keefe SJ (2019). The association between dietary fibre deficiency and high-income lifestyle-associated diseases: Burkitt’s hypothesis revisited. Lancet Gastroenterol Hepatol.

[28] Musil D, Kaletova M, Herman J (2011). Age, body mass index and severity of primary chronic venous disease. Biomed Pap Med Fac Univ Palacky Olomouc Czech Repub.

[29] van Rij AM, De Alwis CS, Jiang P (2008). Obesity and impaired venous function. Eur J Vasc Endovasc Surg.

[30] Willenberg T, Schumacher A, Amann-Vesti B (2010). Impact of obesity on venous hemodynamics of the lower limbs. J Vasc Surg.

[31] Barber GA, Weller CD, Gibson SJ (2018). Effects and associations of nutrition in patients with venous leg ulcers: a systematic review. J Adv Nurs.

[32] Meulendijks AM, de Vries FMC, van Dooren AA, Schuurmans MJ, Neumann HAM (2019). A systematic review on risk factors in developing a first‐time Venous Leg Ulcer. J Eur Acad Dermatol Venereol.

[33] Álvarez-Fernández LJ, Lozano F, Marinello-Roura J, Masegosa-Medina JA (2008). Encuesta epidemiológica sobre la insuficiencia venosa crónica en España: estudio DETECT-IVC 2006. Angiologia.

[34] Fragkouli K, Mitselou A, Boumba VA, Siozios G, Vougiouklakis GT, Vougiouklakis T (2012). Unusual death due to a bleeding from a varicose vein: a case report. BMC Res Notes.

[35] Byard RW, Gilbert JD (2007). The Incidence and Characteristic Features of Fatal Hemorrhage Due to Ruptured Varicose Veins: A 10-Year Autopsy Study. Am J Forensic Med Pathol.

[36] Serra R, Ielapi N, Bevacqua E (2018). Haemorrhage from varicose veins and varicose ulceration: A systematic review. Int Wound J.

[37] Lima DC (2019). Varicose veins and occupational health: symptoms, treatment and prevention. Rev Bras Med Trab..

[38] Jung S, Kim Y, Kang D, Kim SY, Kim I, Kim EM (2020). Distribution of working position among workers with varicose veins based on the National Health Insurance and National Employment Insurance data. Ann Occup Environ Med.

[39] Davies AH (2019). The seriousness of chronic venous disease: a review of real-world evidence. Adv Ther.

