# Supplementary material for: Factors associated with chronic venous disease: study in 1,136 patients treated for varicose veins of the lower limbs in a specialized clinic
Source: J Vasc Bras. 2022 Oct 10;21:e20220051. doi: 10.1590/1677-5449.202200513 (PMC9565676; doi:10.1590/1677-5449.202200513)
Supplement: Translation

# Translation: Factores asociados con la enfermedad venosa crónica: estudio en 1.136 pacientes tratados por várices de miembros inferiores en una clínica especializada en Colombia

**DOI:** 10.1590/1677-5449.202200512

**Authors:** Martha Ofelia Correa Posada, Laura Maria Contreras Correa, John Fernando García Vélez

**Affiliations:** 1 Universidad de Antioquia (U de A), Medellín, Antioquia, Colombia.; 2 Universidad CES (U CES), Medellín, Antioquia, Colombia.; 3 General Practitioner in Vía Vascular Medical, Medellín, Antioquia, Colombia.

**Keywords:** várices, epidemiologia, factores de riesgo

## Abstract

**Contexto:**

Las várices son una condición altamente prevalente en la población general, generando motivos variables de consulta que pueden alterar la calidad de vida del paciente, con prevalencia y factores asociados variando en diferentes series.

**Objetivos:**

Describir el perfil epidemiológico de pacientes que consultaron por várices, evaluando los principales síntomas y las variables asociadas.

**Métodos:**

Entre 2019 y 2020, se evaluaron a 1.136 pacientes que asistieron a una consulta de cirugía vascular en un centro ambulatorio especializado. Se registraron variables demográficas, sintomatología, complicaciones y factores asociados, como índice de masa corporal, paridad e historia familiar.

**Resultados:**

Se evaluaron 1.136 pacientes (79,8% mujeres y 20,2% hombres), con edad media de 53,51 años. La presencia de síntomas fue similar en hombres y mujeres; las complicaciones más frecuentes fueron úlcera, varicorragia y trombosis venosa superficial. La mayoría de los pacientes presentaba CEAP 1, 2 o 3 (n = 909), y más de la mitad tenía sobrepeso u obesidad (n = 679), con predominancia de los clasificados como C4. Sesenta y nueve por ciento tenían historia familiar positiva de várices. No hubo diferencia entre la severidad de las varices y el tiempo laborado de pies o sentado, pero hubo mayor presencia de úlcera C5 o C6 en pacientes que permanecían de pies por más de 4 horas.

**Conclusiones:**

La descripción de las características de la población con várices ayuda a entender la enfermedad y a concentrar los esfuerzos en aquellos más susceptibles. Los resultados de esta investigación son semejantes a los de otras poblaciones.

## INTRODUCCIÓN

Las várices son una condición frecuente en la consulta de atención primaria y una de las más atendidas por los cirujanos vasculares en el mundo, produciendo una variedad de síntomas importantes y complicaciones a largo plazo que van en detrimento de la salud y la calidad de vida, lo cual aumenta igualmente el gasto en salud^1^. El conocimiento de la población con várices hace más fácil el entendimiento de la enfermedad y la educación al paciente, lo cual ayuda a enfocar los esfuerzos en la atención temprana e influir en su desenlace^2^. El objetivo de este trabajo fue describir el perfil epidemiológico de los pacientes que consultaron por várices, evaluando los principales síntomas y las variables asociadas.

## METODOLOGÍA

Estudio prospectivo y descriptivo en el que se tomaron los datos de pacientes que asistieron a una consulta de cirugía vascular en un centro de enfermedades vasculares en la ciudad de Medellín, Colombia, en el período comprendido entre junio de 2019 y diciembre de 2020. A los pacientes que consultaron por várices, se les indagaba por variables relacionadas con la enfermedad venosa, y las respuestas se descargaron en una base de datos previamente constituida en Excel durante la consulta; en ella se incluyeron variables demográficas, antecedentes patológicos relacionados, sintomatología, tratamientos previos, complicaciones, historia familiar y hábitos y variables clínicas como la severidad según la clasificación *clinical, etiological, anatomical, pathophysiological* (CEAP) y el índice de masa corporal (IMC). Es de anotar que en el 2020 se presentó confinamiento por la pandemia derivada del COVID-19, por lo cual se enlenteció la recolección de los datos, y se tuvieron en cuenta solo las consultas presenciales que se pudieron realizar en este período.

Se siguieron los lineamientos en cuanto al manejo de datos personales y políticas de ética de la institución, siendo calificada como investigación sin riesgo. Los datos fueron procesados en SPSS versión 22, utilizando frecuencias y porcentajes para las variables categóricas y medias y desviación estándar (DE) para las variables cuantitativas. Se utilizo la prueba de chi-cuadrado para buscar relación entre las variables categóricas, teniendo en cuenta una p menor 0.05 para significancia estadística.

Este estudio se realizó según la Declaración de Helsinki y la ley colombiana para ética e investigación: Resolución 8430 de 1993, expedida por el Ministerio de Salud y Protección Social de Colombia. El protocolo fue aprobado por el comité de ética de la institución realizadora (acta número 002 de 21 de enero del 2019).

## RESULTADOS

El total de pacientes evaluados fue de 1.136 pacientes, 911 mujeres (80%) y 225 hombres (20%). La media de la edad fue de 53.51 años (DE±12,53; rango 15-98 años) para la población general, 54 (DE±12,25) años para mujeres, y 51,32 (DE±13,39) para los hombres, sin diferencia de edad entre los dos grupos (p = 0,127). La distribución por género de la edad y otras características pueden verse en la Tabla 1.

**Tabla 1:** Variables asociadas a las várices según el género.

**Variable**	**Mujer**		**Hombre**		**Total**	
	**n (911)**	**%**	**n (225)**	**%**	**n = 1.136 (%)**	**Valor de p**
**Edad (años)**						0,045
15-35	76	8,3	31	13,8	107 (9,4)	
36-50	275	30,2	71	31,6	346 (30,5)	
51-60	282	31,0	68	30,2	350 (30,8)	
>60	278	30,5	55	24,4	333 (29,3)	
**Clasificación clínica del CEAP**						0,000
0	7	0,8	6	2,7	13 (1,1)	
1	217	23,8	15	6,7	232 (20,4)	
2	429	47,1	108	48	537 (47,3)	
3	161	17,6	38	16,9	199 (17,5)	
4	38	4,2	26	11,6	64 (5,6)	
5	39	4,3	22	9.8	61 (5,5)	
6	20	2,2	10	4,4	30 (2,6)	
**Síntomas**						
Edema	433	47,5	94	41,8	527 (46,4)	0,121
Pesadez	419	46	86	38,2	505 (44,5)	0,36
Cansancio	563	61,8	125	55,6	688 (60,6)	0,086
Ardor	514	56,4	83	36,9	597 (52,6)	0,000
Prurito	367	40,3	80	35,6	447 (39,3)	0,193
Dolor	598	65,6	123	54,7	721 (63,5)	0,002
Calambres	402	44,1	86	38,2	488 (43)	0,196
**IMC(kg/m^2^)**						0,103
Bajo peso (< 18,5)	6	0,7	4	1,8	10 (0,9)	
Normal (18,5-24,99)	322	35,3	77	34,2	399 (35,1)	
Sobrepeso (25-29,99)	347	38,1	98	43,6	445 (39,2)	
Obesidad (> 30)	236	26	46	20,4	282 (24,8)	
**Factor asociado**						
Diabetes	56	6,1	10	4,4	66 (5,8)	0,328
Hipotiroidismo	166	18,2	8	3,6	174 (15,3)	0,000
Fumador	72	8	30	13,3	102 (9)	0,011
Constipación	134	14,7	10	4,4	144 (12,7)	0,000

CEAP = *clinical, etiological, anatomical, pathophysiological*; IMC = índice de masa corporal.

Además, 891 (78,33%) pacientes se encontraron dentro de los grupos del 2 al 6 de la clasificación clínica del CEAP. La enfermedad C4 o mayor fue más frecuente en hombres que en mujeres, mientras que la C2 y C3 fue muy similar en ambos sexos. La distribución de los síntomas fue parecida en hombres y mujeres, encontrando solo diferencias para la presencia de ardor y dolor, que predominaron en las mujeres. El dolor fue el síntoma más referido en ambos géneros, seguido de cansancio y ardor. De los síntomas evaluados, se encontraron diferencias en la percepción por género únicamente para el ardor y el dolor, que predominaron en mujeres (p < 0,05).

Se encontró una baja prevalencia de diabetes, hipotiroidismo, constipación y fumadores en la población estudiada, pero llama la atención la alta prevalencia de sobrepeso y obesidad (64%) en esta población. Sin embargo, no se encontró diferencia al comparar el estado nutricional entre hombres y mujeres, pero sí al compararlo según severidad, siendo mayor la presencia de grados C4 o mayores en los pacientes con IMC por encima de 25 kg/m^2^.

El 69% (782 pacientes) tenían antecedente de por lo menos uno de los dos padres con afectación por várices, y el 13,11% (149 pacientes) tenían afectación de ambos padres. Además, 212 pacientes (19%) habían sufrido una o más complicaciones, de estas la úlcera fue la más común, presentándose en 91 pacientes (8%), seguida de la varicorragia 22 (2,1%). También se observó que 83 (7,9%) pacientes tenían historia de trombosis venosa superficial, 19 (1,08%) tenían historia de trombosis venosa profunda, y 11 (1,04%) tuvieron linfedema asociado. Solo el 2,6% tenían úlcera activa en el momento de la evaluación.

No se encontraron diferencias en la distribución de los síntomas por género y grupos de edad, excepto para el cansancio, que predominó en los mayores de 35 años en ambos grupos (mujeres: p = 0,003; hombres: p = 0,011). En las mujeres se encontraron diferencias en la distribución del peso por grupos de edad, predominando el sobrepeso y la obesidad por encima de los 35 años, mientras que en hombres esta distribución fue similar para todos los grupos de edad (Tabla 2).

**Tabla 2:** Distribución de los síntomas y estado nutricional según los grupos de edad.

**Síntoma**	**Grupo de edad**				
	**15-35 años**	**36-50 años**	**51-60 años**	**>60 años**	**Valor de p**
**Mujeres**					
Edema	39 (4,3%)	131 (14,4%)	136 (14,6%)	127 (13,9%)	0,832
Pesadez	43 (4,7%)	137 (15,0%)	118 (13,0%)	121 (13,3%)	0,053
Cansancio	61 (6,7%)	174 (19,1%)	166 (18,2%)	162 (17,8%)	0,003
Ardor	44 (4,8%)	156 (17,1%)	162 (17,8%)	152 (16,7%)	0,909
Prurito	36 (4,0%)	116 (12,7%)	110 (12,2%)	105 (11,5%)	0,404
Dolor	53 (5,8%)	176 (19,3%)	188 (20,6%)	181(19,9%)	0,787
Calambres	38 (4,2%)	124 (13,6%)	120 (13,2%)	120 (13,2%)	0,670
**Hombres**					
Edema	14 (6,2%)	32 (14,2)	26 (11,6%)	22 (9,8%)	0,829
Pesadez	15 (6,7%)	33 (14,7%)	22 (9,8%)	16 (87,1%)	0,096
Cansancio	23 (10,2%)	45 (20,0%)	34 (15,1%)	23 (10,2%)	0,011
Ardor	15 (6,7%)	26 (11,6%)	24 (10,7%)	18 (8,0%)	0,523
Prurito	16 (7,1%)	26 (11,6%)	21 (9,3%)	17 (7,6%)	0,196
Dolor	17 (7,6%)	41 (18,2%)	33 (14,7%)	32 (14,2%)	0,664
Calambres	12 (5,3%)	28 (12,4%)	25 (11,1%)	21 (9,3%)	0,991
**Mujeres**					0,001
Bajo peso (< 18,5 kg/m^2^)	0 (0,0%)	0 (0,0%)	2 (0,2%)	4 (0,4%)	
Normal (18,5-24,9 kg/m^2^)	44 (4,8%)	106 (11,6%)	86 (9,4%)	86 (9,4%)	
Sobrepeso (25-29,9 kg/m^2^)	18 (2,0%)	108 (11,9%)	113 (12,4%)	108 (11,9%)	
Obesidad (> 30 kg/m^2^)	14 (1,5%)	61 (6,7%)	81 (8,9%)	80 (8,8%)	
**Hombres**					0,072
Bajo peso (< 18,5 kg/m^2^)	3 (1,3%)	0 (0,0%)	1 (0,4%)	0 (0,0%)	
Normal (18,5-24,9 kg/m^2^)	11 (4,9%)	24 (10,7%)	21 (9,3%)	21 (9,3%)	
Sobrepeso (25-29,9 kg/m^2^)	12 (5,3%)	29 (12,9%)	32 (14,2%)	25 (11,1%)	
Obesidad (>30 kg/m^2^)	5 (2,2%)	18 (8,0%)	14 (6,2%)	9 (4,0%)	

En cuanto a la distribución según el grado de severidad clínica, la mayoría de los pacientes con enfermedad avanzada se encontraron por encima de los 50 años, y la mayoría de los pacientes consultaban en grado C2, seguido por el C1 en todos los grupos de edad (Tabla 3).

**Tabla 3:** Distribución de la edad y síntomas según la clasificación clínica del CEAP.

**Variable**	**Clasificación clínica del CEAP**							
	**C0**	**C1**	**C2**	**C3**	**C4**	**C5**	**C6**	**Valor de p**
Edad (años)								0,000
15-35	2 (1,9%)	14 (13,1%)	65 (60,7%)	15 (14%)	5 (4,7%)	2 (1,9%)	4 (3,7%)	
36-50	4 (1,2%)	76 (22%)	164 (47,4%)	72 (20,8%)	12 (3,5%)	11 (3,2%)	7 (2,0%)	
51-60	6 (1,7%)	75 (21,4%)	163 (43,9%)	64 (18,3%)	17 (4,9%)	16 (4,6%)	9 (2,6%)	
> 60	1 (0,3%)	67 (20,1%)	145 (43,5%)	48 (14,4%)	30 (9,0%)	32 (9,6%)	10 (3.0%)	
**Síntomas**								
Edema	4 (0,8%)	82 (15,6%)	213 (40,4%)	140 (26,6%)	35 (6,5%)	19 (3,6%)	1 (3,6%)	0,000
Pesadez	7 (1,4%)	101 (20,0%)	231 (45,7%)	99 (19,6%)	24 (4,8%)	26 (5,1%)	17 (3,4%)	0,380
Cansancio	8 (1,2%)	131 (19,0%)	339 (49,3%)	126 (18,3%)	32 (4,7%)	33 (4,8%)	19 (2,8%)	0,245
Ardor	7 (1,2%)	121 (20,3%)	289 (48,4%)	17,9 (4,6%)	28 (4,7%)	21 (3,5%)	24 (4,0%)	0,003
Prurito	3 (3,7%)	81 (18,1%)	206 (46,1%)	84 (18,8%)	32 (7,2%)	29 (6,5%)	12 (2,7%)	0,172
Dolor	8 (1,1%)	137 (19,0%)	341 (47,3%)	135 (18,7%)	42 (5,8%)	31 (4,3%)	27 (3,7%)	0,009
Calambres	0 (0,0%)	102 (20,9%)	237 (48,6%)	92 (18,9%)	21 (4,3%)	20 (4,1%)	16 (3,3%)	0,007

CEAP = *clinical, etiological, anatomical, pathophysiological*.

La mayoría de los pacientes sintomáticos se encontraron en la clasificación C2, predominando dolor, cansancio y calambres en este grupo de pacientes. En los pacientes con C0 y C6, los síntomas más prevalentes fueron cansancio, pesadez y ardor. Solo el ardor y el dolor tuvieron diferencias estadísticamente significativas según el género. En la Tabla 3 se pueden observar las relaciones entre los grados de C y los factores asociados a las várices

El promedio de embarazos fue de 2,72 (DE±2,1). Además, 91 mujeres (8%) no habían tenido ningún embarazo al momento del estudio, 408 (36%) tenían entre uno y dos embarazos, y 412 mujeres (36,3%) tuvieron más de tres embarazos. Al comparar el número de embarazos y la severidad según CEAP, no se encontró ninguna diferencia estadísticamente significativa (p = 0,196), sin embargo, fue mayor el número de mujeres con enfermedad avanzada (C4 o más) en las que tenían 3 o más embarazos (58 *versus* 34) (Tabla 3).

Un total 575 (50,6%) pacientes ejercían algún tipo de labor remunerada. El promedio de horas laboradas sentados, basado en una jornada de 8 horas, fue de 4,92 (DE±2,49) y de 6,85 (DE±1,88) para las horas laboradas de pies. Al agrupar las horas laboradas, no se encontraron diferencias entre la severidad de las várices y el tiempo laborado de pies o sentado; sin embargo, la presencia de úlcera C5 o C6 fue mayor en los pacientes que permanecían más de 4 horas de pies.

## DISCUSIÓN

Este artículo se basa en los resultados de la consulta general del cirujano vascular, lo que puede ser una limitación. Sin embargo, centra los resultados en los factores asociados a la enfermedad venosa, algo útil en la práctica diaria.

Aunque la diferencia entre hombres y mujeres con várices puede variar según los criterios de inclusión y la metodología entre los diferentes estudios, la mayoría de los reportes muestran una mayor frecuencia en mujeres que hombres, como lo encontrado en esta serie (80% *versus* 20%), aunque puede ser un sesgo de selección, ya que puede reflejar una mayoría de mujeres que consultan por várices^3^^-^5. Se encontró también mayor proporción de mujeres que consultan en C1 comparado con los hombres (217 *versus* 15), debido tal vez al impacto cosmético en las mujeres, que aumentaría la consulta en estadios tempranos. Sin embargo, también hay una mayor proporción de síntomas en las mujeres con C1 comparado con los hombres en la misma categoría. Otros estudios han mostrado una mayor proporción de hombres con cambios tróficos visibles y enfermedad avanzada, al igual que en esta serie^6^^,^^7^.

Varios autores han mostrado la progresión de la enfermedad con el tiempo^8^^-^10, con baja prevalencia en los menores de 30 años, al igual que los datos de este estudio, donde la mayoría de los pacientes se encuentran por encima de los 35 años en ambos géneros. La severidad en los estadios C4-C6 presenta un aumento relacionado con la edad, esto concuerda con que la enfermedad venosa requiere tiempo para establecer un daño más severo y que puede ser necesario la intervención temprana para disminuir el deterioro de la salud vascular^11^.

Hay una gran variedad de síntomas que están presentes en la enfermedad venosa pero no son exclusivos de esta entidad, esto hace que el diagnóstico diferencial en ocasiones sea complejo. Al analizar por la clasificación C, se tuvo en cuenta la C0, en la cual se pueden encontrar síntomas pero sin signos objetivos de várices, encontrando solo un 1,1% de los pacientes dentro de esta clasificación, siendo un porcentaje muy bajo, comparado con estudios poblacionales como el *Vein Consult*, en el cual la prevalencia de C0 fue del 20%^12^. Esto puede deberse a que muchos de los pacientes que llegan a la consulta vascular ya han sido evaluados previamente por médicos de atención primaria, los cuales, al no ver signos externos de várices, pueden no enviar al paciente a la consulta especializada.

El estudio de Edimburgo mostró diferencias entre síntomas como edema, cansancio y prurito, los cuales fueron mayores en las mujeres jóvenes que en los hombres^13^. Sin embargo, en este estudio se encontró una distribución muy similar de los síntomas entre los diferentes grupos de edad según el género, lo cual puede deberse a un mayor número de pacientes con várices C2 o mayores, que se pueden asociar a mayor presencia de síntomas, y a la demora en el acceso a cirugía vascular como subespecialidad en el sistema de salud, que hace que los pacientes lleguen más sintomáticos.

El embarazo se ha encontrado como factor de riesgo para el desarrollo de várices, puede ser por los cambios hemodinámicos secundarios al aumento de la presión intrabdominal o por la presencia de alto contenido hormonal circulante^14^. Un metaanálisis que busco determinar si la historia de embarazos se asociaba con el desarrollo de varices, encontró un aumento del riesgo de sufrir várices de un 82% [*odds ratio* (OR) = 1,82; intervalo de confianza del 95% (IC95%) = 1,433-2,33] en las mujeres que han tenido embarazos comparado con las que no^15^. Lee et al.^10^, al evaluar los factores asociados a la progresión de la enfermedad venosa en la población del estudio de Edimburgo, tampoco pudo encontrar una asociación entre severidad y número de gestaciones. Un estudio de prevalencia en la población colombiana de un resguardo indígena mostró que edad, sexo femenino, paridad (más de cuatro embarazos), contenido de grasa en el muslo y talla alta se asociaron a telangiectasias, pero no encontraron asociación con estados más avanzados de las várices^16^. En esta serie no se encontró relación entre número de gestaciones y severidad de la enfermedad.

Diversos reportes han mostrado una historia familiar de várices entre las personas afectadas, con reportes variando entre el 42% y el 85%, lo que concuerda con los datos de este estudio, en el cual el 69% de los pacientes tenían por lo menos uno de los padres con afectación^17^^-^19. Algunos estudios han logrado identificar genes relacionados con la enfermedad venosa que pueden explicar la influencia de la presencia de familiares en primer grado con la aparición de los trastornos venosos^20^^-^22.

No hay consistencia en la literatura acerca de si el tabaquismo juega o no un papel importante en el desarrollo de várices. Al igual que en este estudio, algunos no encuentran esta relación^23^^,^^24^; sin embargo, recientemente, Yuan et al. reportan que el cigarrillo es un factor de riesgo independiente (OR = 2.53; IC95% = 1.15, 5.58)^25^, mientras que otros encuentran que pudiera haber una asociación positiva en la predisposición genética a fumar con el riesgo de várices (OR = 1,12; IC95% = 1,04–1,22; p = 0,004)^26^. Desde la hipótesis de Burkit sobre el tránsito intestinal lento y su asociación con diferentes enfermedades, la constipación aparece como un factor relacionado por algunos autores para el desarrollo de várices por el aumento de la presión intrabdominal^27^. Sin embargo, no se pudo probar una asociación con este factor ya que la prevalencia en esta población fue baja.

La obesidad también se ha señalado con una correlación positiva entre IMC y desarrollo y severidad de las várices, con una mayor severidad en los pacientes obesos, esto debido al aumento de la presión intrabdominal por el efecto de masa de la grasa contenida sobre las venas de la pelvis, lo que puede afectar el retorno venoso, cambios que también pueden aplicarse al aumento de la presión intrabdominal por el crecimiento del útero durante el embarazo^28^^-^30. En este estudio se pudo encontrar diferencias entre la presencia de varices en los diferentes grados de C con respecto al estado nutricional, encontrando una mayor proporción de pacientes con úlcera activa o historia de úlcera venosa entre los pacientes con sobrepeso u obesos comparado con los no obesos (54% *versus* 2,28%), hallazgos similares a otras series donde un mayor IMC se asocia a desarrollo de úlceras^31^^,^^32^.

La prevalencia de la úlcera activa en la población estudiada fue del 2,6%, lo cual concuerda con la mayoría de las series^4^^,^^33^. La varicorragia es una complicación que si no se maneja adecuadamente incluso puede ser mortal, principalmente en casos donde no se procede a primeros auxilios adecuados, se ha encontrado que puede ser la causa de muerte en el 0,01% de las autopsias^34^^-^36. La incidencia de esta complicación es difícil de establecer en la literatura, posiblemente por su baja prevalencia, en este estudio solo 22 pacientes habían tenido algún episodio.

No se encontró relación entre el tiempo laborado de pies o sentado con la severidad de las várices, pero no se tuvo en cuenta el tiempo de exposición a este factor, o si era una posición estática o dinámica. La falta de uniformidad en estos criterios entre los diferentes estudios ha hecho difícil encontrar una relación entre ocupación y desarrollo de várices, además de la existencia de múltiples factores que pueden influir en su aparición. Sin embargo, algunos reportes sugieren que puede haber una asociación positiva entre las posiciones prolongadas durante mucho tiempo y la aparición de várices, y que el cambio positivo en los estilos de vida puede ayudar a disminuir la severidad de los síntomas y mejorar la calidad de vida en el trabajo^37^^-^39.

Como conclusión, los resultados de este estudio muestran una distribución de la enfermedad venosa entre la población evaluada muy similar a la encontrada en otras series, apoyan la progresión de la severidad clínica con la edad y el IMC, así como una mayor frecuencia de historia familiar de várices en los pacientes evaluados. El conocimiento de las características de la población favorece la orientación a los pacientes sobre la historia natural de la enfermedad y también mejora la optimización de recursos al establecer prioridades con base en la existencia de factores de riesgo o complicaciones derivadas, lo que puede repercutir en una mejor calidad de vida y una disminución del gasto en salud.
